# The Association Between Apolipoprotein E and Functional Outcome After Traumatic Brain Injury: A Meta-Analysis

**DOI:** 10.1097/MD.0000000000002028

**Published:** 2015-11-20

**Authors:** Lizhuo Li, Yijun Bao, Songbai He, Gang Wang, Yanlei Guan, Dexuan Ma, Rile Wu, Pengfei Wang, Xiaolong Huang, Shanwei Tao, Qiwen Liu, Yunjie Wang, Jingyun Yang

**Affiliations:** From the Department of Critical Care and Emergency Medicine, The Affiliated Hospital of Hainan Medical University, Haikou, Hainan (LL); Emergency Department, Shengjing Hospital of China Medical University (LL, SH, GW, QL); Department of Neurosurgery, The First Hospital of China Medical University, Shenyang, Liaoning (YB, YG, PW, XH, ST, YW); Department of Neurosurgery, Huashan Hospital, Fudan University, Shanghai (DM); Department of Occupational and Environmental Health, School of Public Health, Shenyang Medical College, Shenyang, Liaoning, China (RW); Rush Alzheimer's Disease Center (JY); and Department of Neurological Sciences, Rush University Medical Center, Chicago, Illinois (JY).

## Abstract

Traumatic brain injury (TBI) is a leading cause of death and disability. Previous studies have investigated the association of apolipoprotein E (APOE) ε4 with functional outcome after TBI and reported inconsistent results.

The purpose of this study was to perform a systematic literature search and conduct meta-analyses to examine whether APOE ε4 is associated with poorer functional outcome in patients with TBI.

We performed a systematic literature search in PubMed, Cochrane Library, Embase, Google Scholar, and HuGE.

The eligibility criteria of this study included the following: Patients had TBI; the studies reported APOE genotype data or provided odds ratios (ORs) and the corresponding 95% confidence intervals (CIs); the functional outcome was assessed using the Glasgow Outcome Scale (GOS) or the Glasgow Outcome Scale Extended (GOSE); and patients were followed for at least 3 months after TBI.

In all meta-analyses, we used random-effects models to calculate the odds ratio as a measure of association. We examined the association of APOE ε4 with functional outcome at different time points after TBI.

A total of 12 studies met the eligibility criteria and were included in the meta-analyses. We did not find a significant association between APOE ε4 and functional outcome at 6 (*P* = 0.23), 12 (*P* = 0.44), and 24 months (*P* = 0.85) after TBI. However, APOE ε4 was associated with an increased risk of unfavorable long-term (≥6 months) functional outcome after TBI (OR = 1.36, 95% CI: 1.07–1.74, *P* = 0.01).

Limitations of this study include The sample size was limited; the initial severity of TBI varied within and across studies; we could not control for potential confounding factors, such as age at injury and sex; a meta-analysis of the genotype dosage effect was not feasible; and we could not examine the association with specific factors such as neurobehavioral or specific cognitive functions.

Our meta-analysis indicates APOE ε4 is associated with the long-term functional outcome of patients with TBI. Future studies that control for confounding factors, with large sample sizes and more homogeneous initial TBI severity levels, are needed to validate the findings from this study.

## Abstract 2

Supplemental Digital Content is available in the text

## INTRODUCTION

Traumatic brain injury (TBI) results from a violent external mechanical force to the head or body, such as a motor vehicle crash, a sports injury, or a simple fall to the ground, that causes brain dysfunction. TBI can result in various devastating symptoms and morbidity or mortality, and is the leading cause of death and disability among persons aged 44 or younger, with approximately 52,000 deaths each year in the United States.^1^ More than 5.3 million Americans, 2% of the US population, suffer from disability due to TBI.^2^

The apolipoprotein E (APOE) gene is located on chromosome 19, close to the Apo C-I and C-II genes. APOE is a major component of very low-density lipoproteins (VLDLs), which plays an essential role in maintaining normal blood cholesterol level by removing excessive cholesterol to the liver for processing.^3^ APOE can mediate lipoprotein binding to the LDL receptors and regulate cholesterol metabolism and repair of cell membrane and growth of neurites after injury in the central nervous system (CNS).^4^ APOE has 3 polymorphic alleles, ε2 (cys112, cys158), ε3 (cys112, arg158), and ε4 (arg112, arg158). The ε4 isoform has been implicated in atherosclerosis, ischemic cerebrovascular disease, impaired cognitive function, and late-onset Alzheimer's disease (AD).^5–7^

Recovery after TBI is heterogeneous and depends on the age of the patient, and the nature, location, and extent of the injury.^8^ Known predictors account for only a limited percentage of the variation in outcomes.^9^ Recent studies indicated that genetic variants, such as the APOE polymorphism, may also contribute to the severity and outcome of TBI.^10,11^ However, the results and conclusion of these studies are not consistent, and the relation between the APOE genotype and TBI recovery remains unclear, partly due to the variable methods and limited sample sizes.

To the best of our knowledge, 2 meta-analyses have been conducted to address this inconsistency.^12,13^ The first meta-analysis was published in 2008, and included studies published before October 2007.^12^ Many new studies that further examined the association of APOE with functional outcome after TBI have been published since then. Another meta-analysis,^13^ published in 2014, examined the association of APOE with prognosis of TBI, but had several limitations: in the study, the definition of prognosis was not clearly defined such that it is unclear at what time point after TBI the authors performed a meta-analysis of the prognosis; the authors analyzed overlapping data; the authors utilized binary prognosis (good vs bad) as the main outcome of interest. During a literature search, they might have excluded studies that provided quantitative functional outcome of patients with TBI; and we suspect that the calculation of the odds ratios (ORs) was incorrect for some studies.

Therefore, we performed this updated meta-analysis to analyze the association of APOE with the functional outcome of patients with TBI at different time points after TBI, which has not been published before. Moreover, we included studies that provided quantitative results for functional outcome measured with the Glasgow Outcome Scale (GOS) or the Glasgow Outcome Scale Extended (GOSE), as well as studies that provided results for binary prognosis.

## METHOD

### Eligibility Criteria

We used the following inclusion criteria to determine study eligibility: patients had TBI; the studies reported APOE genotype data (APOE ε4^+^ vs ε4^−^), or provided ORs and the corresponding 95% confidence intervals (CIs) regarding the association of APOE with the functional outcome of patients with TBI; the function outcome of the patients was assessed using the GOS or the GOSE; and the patients were followed for at least 3 months after TBI. If multiple studies used overlapping datasets, we chose the one with the larger sample size.

### Search Strategy

Two authors (YB and JY) performed an extensive literature search in PubMed, Cochrane Library, Embase, Google Scholar, and HuGE for papers published before March 13, 2015. The keywords used in the literature search are provided in the online supplementary file (Supplementary file, Keywords used in the literature search, http://links.lww.com/MD/A521).

All potentially relevant publications were retrieved and evaluated for study eligibility. The references in all relevant studies were also searched for studies that might have been missed during the literature search. The 2 authors performed the literature search independently. The search was limited to studies published in English. Any disagreement was resolved by group discussion. No efforts were made to contact authors of the included studies for additional data.

### Data Extraction

According to a prespecified protocol for data extraction, 2 authors (LL and JY) independently extracted the following data from the eligible studies: name of the first author, year of publication, participants characteristics including sample size, mean age, distribution of sex, race/country of origin of the participants, time of assessment, measures for outcome assessment, APOE genotype data for patients with different GOS or GOSE scores, or for patients with favorable and unfavorable functional outcomes, or odds ratio and the corresponding 95% CI. For studies that reported APOE data for different GOS or GOSE scores, we dichotomized the GOS or the GOSE score into favorable and unfavorable outcomes, with GOS≥4 or GOSE≥5 being favorable. Two authors (RW and JY) independently assessed the quality of the included studies using Newcastle–Ottawa scale.^14^ Any discrepancies were resolved in a group meeting.

### Data Analysis

ORs were used as a measure of association between the APOE genotype and unfavorable functional outcomes of patients with TBI. In all meta-analyses, we used random-effects models to calculate the OR and the corresponding 95% CI. Between-study heterogeneity was assessed using *I*^2^. Publication bias was evaluated using a funnel plot and Egger's test.

We obtained data for the functional outcome of patients at different time points: 3, 6, 12, 18, 24, and 36 months or beyond after TBI. Meta-analyses were conducted when there were multiple studies for the analysis at each time point after TBI. When a study included a crude OR as well as an adjusted OR, which was obtained after controlling for risk factors, we used the adjusted OR. We evaluated the association of APOE ε4 with the patients’ functional outcome at different time points after TBI as well as with the long-term outcome, defined as the functional outcome at ≥6 months after TBI. For the latter case, we collected the results from all studies that provided data at ≥6 months after TBI. If a study provided outcome assessments multiple times at 6 months or later after TBI, we used the last assessment from that study.

All statistical analyses were performed using Stata 11.2 (StataCorp LP, College Station, TX) and SAS version 9.3 (SAS Institute Inc, Cary, NC). A *P* < 0.05 was considered statistically significant. Since our study used a systematic review and meta-analysis, ethical approval of the study was not required. This study was reported according to the PRISMA guidelines.^15^

## RESULTS

### Study Selection and Characteristics

**FIGURE 1:**
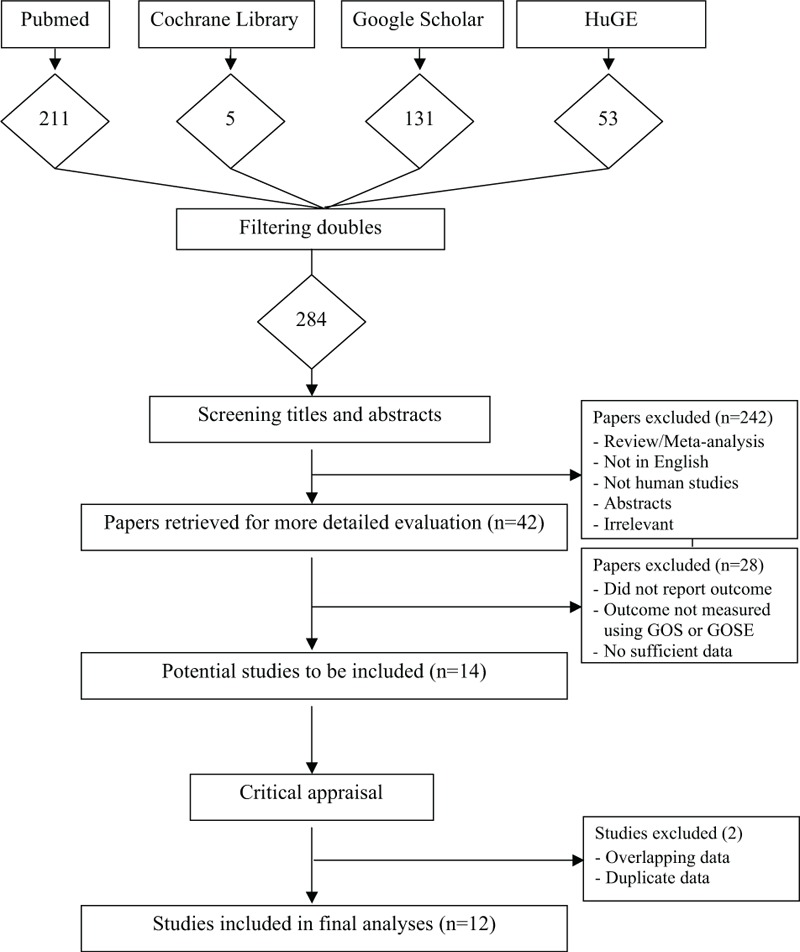
Flow diagram of the selection process of the studies included in the meta-analyses. Please see the Methods section for additional details.

Figure 1 shows the selection of eligible studies included in our meta-analyses. We identified a total of 284 potential publications through our initial search. After the abstracts of these studies were screened, 242 publications were excluded either because they were reviews or meta-analyses, were not about human subjects, were not in English, had been published as abstracts, or were irrelevant. This left 42 studies that were retrieved for more detailed evaluations. We excluded an additional 28 studies because they did not report functional outcome, the outcome was not assessed using the GOS or the GOSE, or there were insufficient data (see the online supplementary data for references, http://links.lww.com/MD/A521). This led to 14 potentially relevant publications for our analysis. We then excluded 2 more studies because they used overlapping or duplicate data,^16,17^ resulting in 12 studies that met the eligibility criteria and were included in our analyses.^18–29^

All included publications had been published since 2003. The sample sizes ranged from 46 to 984 patients (Table 1). Of these 12 studies, 2 studies provided data for outcome at 3 months, 8 studies at 6 months, 3 studies at 12 months, 1 study at 18 months, 2 studies at 24 months, and 1 study at 36 months after TBI. And 12 studies provided data at ≥6 months after TBI. The combined study included 117 patients for evaluating association at 3 months, 1943 at 6 months, 187 at 12 months, 117 at 24 months, and 2628 at ≥6 months after TBI.

**TABLE 1:**
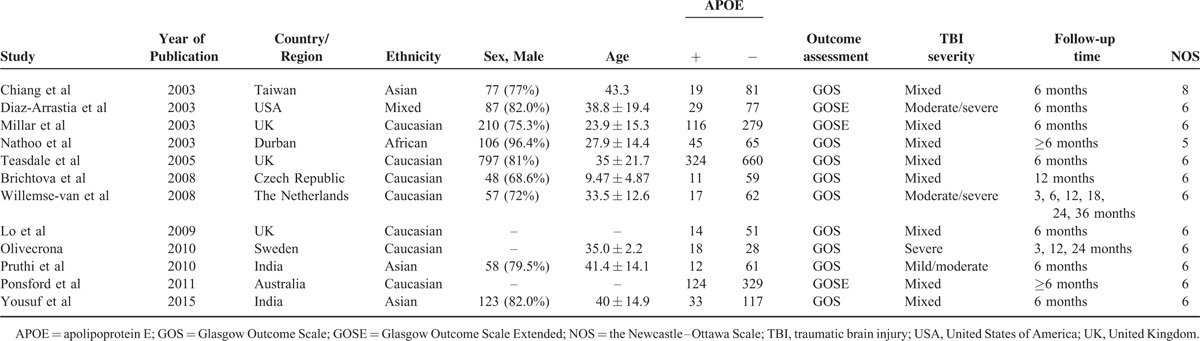
Characteristics of the Included Studies

### Assessment of Publication Bias

**FIGURE 2:**
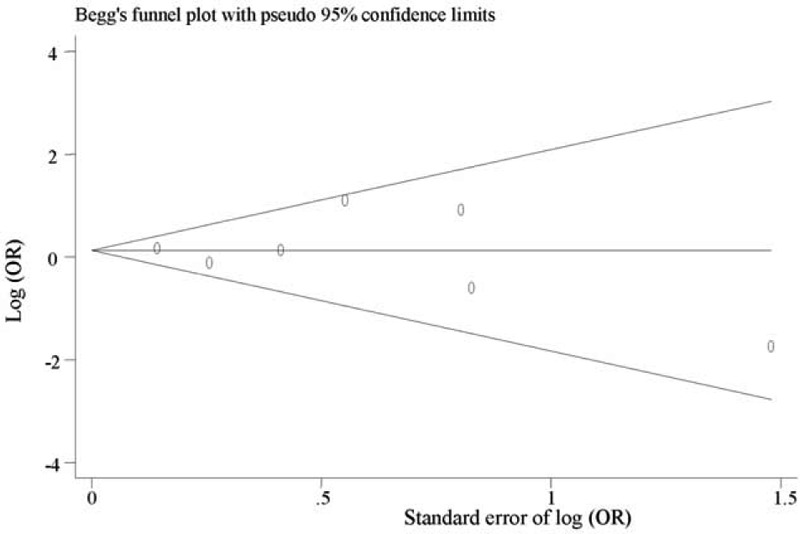
Funnel plot for meta-analysis of the association of APOE ε4 with functional outcome at 6 months after traumatic brain injury. The *x*-axis is the standard error of the log-transformed odds ratio (log [OR]), and the *y*-axis is the log-transformed odds ratio. The horizontal line in the figure represents the overall estimated log-transformed odds ratio. The 2 diagonal lines represent the pseudo 95% confidence limits of the effect estimate. OR = odds ratio.

**FIGURE 3:**
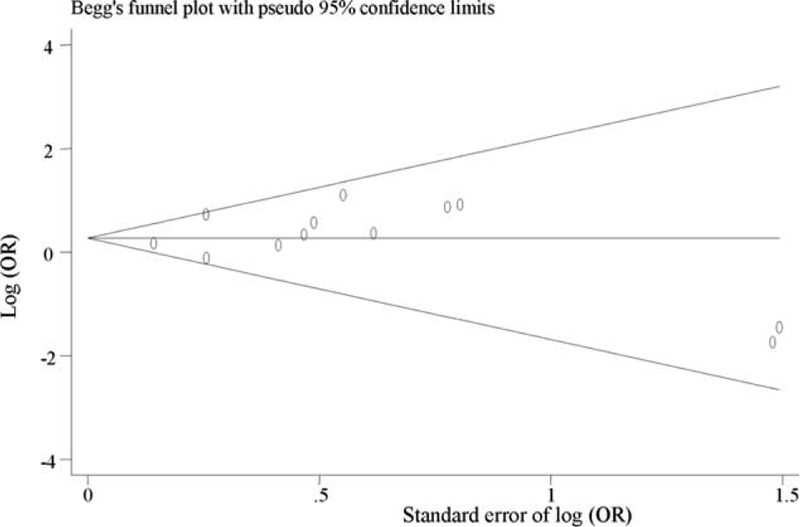
Funnel plot for meta-analysis of the association of APOE ε4 with long-term functional outcome (≥6 months) after traumatic brain injury. The *x*-axis is the standard error of the log-transformed odds ratio (log [OR]), and the *y*-axis is the log-transformed odds ratio. The horizontal line in the figure represents the overall estimated log-transformed odds ratio. The 2 diagonal lines represent the pseudo 95% confidence limits of the effect estimate. OR = odds ratio.

We did not find evidence of publication bias for the meta-analysis of the outcome at 6 months (*P* = 0.57; Fig. 2) or at ≥6 months after TBI (*P* = 0.77, Fig. 3). Assessment of publication bias for the meta-analysis of functional outcome at other time points after TBI is not meaningful because the number of studies included in the corresponding meta-analysis was limited.

### Association of APOE ε4 With Functional Outcome After TBI

**FIGURE 4:**
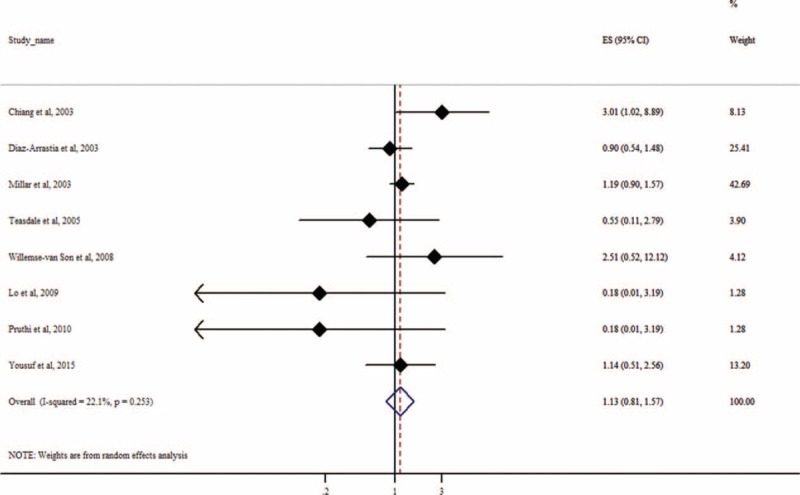
Forest plot for meta-analysis of the association of APOE ε4 with functional outcome at 6 months after traumatic brain injury. Each study is represented by a square whose area is proportional to the weight of the study. The overall effect from meta-analysis is represented by a diamond whose width represents the 95% CI for the estimated odds ratio (OR).

**FIGURE 5:**
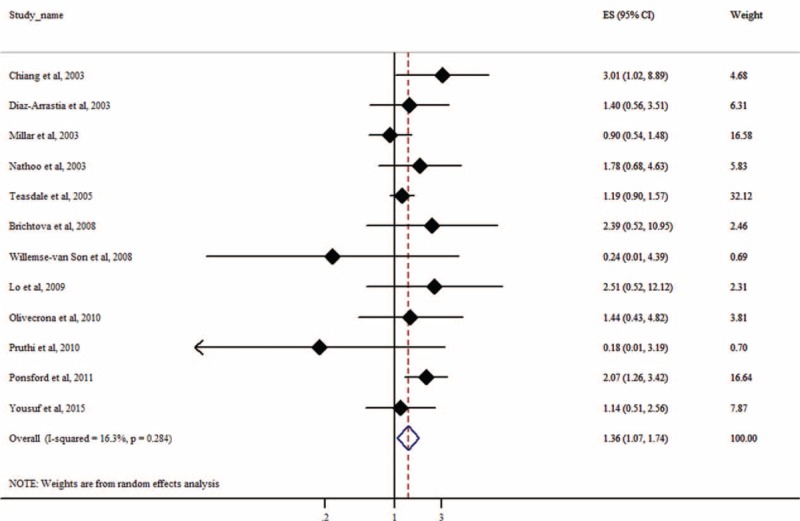
Forest plot for meta-analysis of the association of APOE ε4 with long-term functional outcome (≥6 months) after traumatic brain injury. Each study is represented by a square whose area is proportional to the weight of the study. The overall effect from meta-analysis is represented by a diamond whose width represents the 95% CI for the estimated odds ratio (OR).

We evaluated the association between APOE ε4 and an unfavorable functional outcome at different time points after TBI. Eight studies reported 6-months follow-up data. Only 1 study demonstrated a statistically significant association (*P* = 0.05).^18^ Twelve studies reported ≥6-month follow-up data. Only 2 studies showed a significant association.^18,28^ Our meta-analysis did not find a significant association between APOE ε4 and functional outcome at 6 (*P* = 0.23; Fig. 4), 12 (*P* = 0.44), and 24 months (*P* = 0.85; eTable 1, http://links.lww.com/MD/A521) after TBI. However, APOE ε4 was associated with an increased risk of poor long-term functional outcome (≥6 months) after TBI (OR = 1.36, 95% CI: 1.07–1.74, *P* = 0.01; Fig. 5). The association remained unchanged after we excluded 2 studies^23,25^ that focused on outcomes of TBI in children (OR = 1.33, 95% CI: 1.02–1.74, *P* = 0.04). There was a trend of association at 3 months after TBI (OR = 2.10, 95% CI: 0.91–4.82, *P* = .08; eTable 1, http://links.lww.com/MD/A521). However, these results should be interpreted with caution due to the very limited sample size. One study^24^ provided data for assessing the association between APOE ε4 and functional outcome at 18 and 36 months after TBI, and there was no significant association for both time points (both *P* < 0.34; eTable 1, http://links.lww.com/MD/A521).

To explore sources of heterogeneity, we performed multivariate random-effects meta-regression with covariates including mean age, proportion of male, and ethnicity (Caucasian, Asian, and other). We found that the 3 variables were not a significant source of heterogeneity for meta-analyses of 6-month follow-up and ≥6-month follow-up (all *P*'s > 0.60), which implies that these variables should have limited influence on the validity of our estimation.

### Sensitivity Analysis

The studies included in our meta-analyses were conducted in different ethnicities. To examine whether ethnicity influenced the results, we performed an additional meta-analysis by ethnicity (Asian and Caucasian). We also performed a separate meta-analysis by initial TBI severity (moderate/severe vs mixed) and by excluding studies of low quality (<6 stars).

We found no association between APOE ε4 and functional outcome at 6 months after TBI in the Asian (*P* = 0.57) and Caucasian studies (*P* = 0.37). There was a trend of association with long-term functional outcome (≥6 months) in Caucasians (*P* = 0.08; eTable 2, http://links.lww.com/MD/A521). The result did not reach statistical significance probably due to the reduced sample size. Analysis by initial TBI severity was available for only 3 studies on moderate/severe TBI^19,24,26^ because other studies recruited patients with mixed severity levels. We found no association between APOE ε4 and functional outcome at ≥6 months after TBI for patients with moderate or severe TBI (*P* = 0.50). Again, these results should be interpreted with caution due to the limited sample size. Excluding low-quality studies did not change the results of the association between APOE ε4 and functional outcome at ≥6 months after TBI (OR = 1.35, 95% CI: 1.03–1.76; *P* = 0.03).

## DISCUSSION

In this study, we performed a systematic literature search and conducted updated meta-analyses to examine the association of APOE ε4 with the functional outcome of patients with TBI. We did not find a significant association between APOE ε4 and functional outcome at 6 months after TBI. However, after collecting all available data for long-term functional outcome (≥6 months), we found that APOE ε4 is associated with an increased risk of unfavorable long-term functional outcome. To the best of our knowledge, this updated meta-analysis is the first to examine the association of APOE ε4 with functional outcome at specific time points following TBI.

Two systematic review/meta-analysis papers were published while we were writing the manuscript after the literature search. In the first study, a systematic literature review was conducted, and the authors found that APOE ε4 was associated with poor TBI outcome in 9 (37.5%) out of 24 studies on mild TBI, and in 21 (63.6%) out of 33 studies on severe TBI.^30^ It found that of the 20 studies on the association of APOE ε4 with functional outcome, only 5 (25%) observed a detrimental effect of the ε4 allele. Unlike our study that focused on functional outcome after TBI, this systematic review covered various outcomes following TBI, such as dementia. Moreover, it did not summarize the functional outcome at different time points after TBI. The other meta-analysis paper examined the association of APOE ε4 with pediatric TBI (TBI patients in the age range 3–18 years).^31^ The study included 340 TBI cases for meta-analysis at 6 months, and found that APOE ε4 was associated with increased odds of poor functional outcome at 6 months after TBI (OR = 2.36, 95% CI: 1.26–4.42; *P* = 0.01). Consistent with our result, this study also suggested that, in pediatric TBI patients, the effect of APOE ε4 might be time dependent.

Although some previous studies as well as our meta-analysis indicated a possible association between the APOE ε4 genotype and an unfavorable long-term functional outcome for patients with TBI, in clinic it is still too early to employ APOE genotyping to predict the functional outcome of TBI. Involvement of APOE in multiple brain disorders such as TBI and AD indicates that APOE might play diverse roles in these processes. However, how APOE performs its roles and how its polymorphism influences and/or interacts with clinical management in the central nervous system remain to be elucidated. Interestingly, several studies discussed the importance of calcium homeostasis in healthy and injured brains.^32–34^ It is possible that calcium channel modulators may attenuate the effect of the APOE ε4 allele.^35^ Increased deposition of β-amyloid in patients carrying the ε4 allele may also contribute to the pathological outcome following TBI.^36,37^ In addition to regulation of cholesterol/lipid metabolism, cell growth,^38^ and cell survival,^39^ APOE's involvement in vascular and hematological processes in response to TBI also warrants more investigation. More intensive studies are also needed to translate these findings to the care and management of TBI.

Most previous studies on the functional outcome of TBI used the GOS or the GOSE to assess functional outcome status brain magnetic resonance imaging (MRI) based volumetric analyses may be another important measure for assessing the recovery of patients with TBI. Several studies compared the MRI imaging results during recovery in patients with TBI with or without the APOE ε4 allele,^40–42^ but failed to detect a significant difference. More studies are needed to examine collectively multiple other markers, including neuroradiological markers (eg, computed tomography [CT], MRI, and positron emission tomography [PET]) and biomarkers (eg, CD38, serum S100B, myelin basic protein, neuron-specific endolase, and glial fibrillary acidic protein), to get a better understanding of the relationship between APOE and the functional outcome of patients with TBI.

This study had several limitations: the sample size was limited for many of the included studies, and more studies with larger samples are needed; the initial severity of TBI varied within and across studies; our meta-analyses examined only published data. Due to the lack of original data, we could not control for potential confounding factors, such as age at injury, sex, alcohol consumption, initial TBI severity, and other concomitant trauma. The estimated effect of APOE on functional outcome might be greatly confounded by such factors, which could thus influence the validity of any meta-analysis that uses un-adjusted results. Therefore, future studies on the relationship between APOE and the functional outcome of TBI should take into account these important confounding factors; most studies examined APOE ε4 versus no ε4. Few studies performed gene dosage comparisons (ie, number of ε4) across the full spectrum of genotypes, and a meta-analysis of dosage effect is not feasible; and we analyzed only functional outcome that was assessed using a global measure (the GOS or the GOSE), but did not examine specific factors such as neurobehavioral or specific cognitive functions since these data were scarce. It would be interesting to perform a meta-analysis on whether APOE ε4 is associated with these specific factors when more such data are available.

In summary, in this study, we performed meta-analyses to analyze the association of APOE ε4 with the functional outcome of patients with TBI. We found that APOE ε4 was associated with an increased risk of unfavorable long-term (≥6 months) but not short-term functional outcome (<6 months) after TBI. Future studies with large sample sizes and more homogeneous initial TBI severity that control for important confounding risk factors and/or examine the genotype dosage effect of APOE ε4 on the functional outcome of patients with TBI are needed to validate our findings and to explore additional genetic loci that might affect functional outcome. Future prospective studies should also take into account important comorbid factors, such as diabetes, cardiovascular disorders and hypertension, previous history of head injury, sleep disorders, and family history of dementia to fully elucidate the relationship between APOE ε4 and functional outcome after TBI.
